# Supplementary material for: Are carbon emissions trading and green financial instruments synergistic? -Comprehensive quantitative research based on content analysis
Source: PLoS One. 2024 Mar 7;19(3):e0298601. doi: 10.1371/journal.pone.0298601 (PMC10919601; doi:10.1371/journal.pone.0298601)
Supplement: S1 Data — (ZIP) [file pone.0298601.s001.zip › Data/地级市Z_F工作报告.zip!/┴╔─■/╜⌡╓▌╩╨/└┤╫╘╣1⁄2╓┌║┼í╛┬φ┐╦╩2╛▌═°í┐_2010.docx]

各位代表：

　　现在，我代表市政府向大会报告工作，请予审议，并请市政协各位委员提出意见和建议。

　　一、2009年工作回顾

　　2009年，面对国际金融危机带来的不利影响，我们在省委、省政府和市委的正确领导下，积极应对挑战，战胜各种困难，保持了经济社会的持续快速发展，圆满完成了“四个确保”目标和市十四届人大二次会议确定的各项任务。

　　（一）主要指标实现“两个不低于”，经济保持平稳较快增长

　　预计全市地区生产总值完成790亿元，增长16%，总量位居全省第五位；全社会固定资产投资350亿元，增长40%，增速位居全省第五位；地方财政一般预算收入48亿元，增长36.9%，增速位居全省第三位。保增长目标如期实现，经济实力显著提升。

　　大旱之年农业减产不减收。粮食总产量达到193.9万吨。农林牧渔业总产值实现255.6亿元，增长7.7%。

　　规模以上工业增加值完成269亿元，增长22.3%；工业用电量增长14.1%，增速位居全省第二位。工业经济形势明显好转，企稳回升态势业已形成。

　　社会消费品零售总额完成269.9亿元，增长18.3%。城乡市场繁荣活跃，拉动内需作用进一步增强。

　　实际利用外资3.7亿美元，增长18.4%；引进域外资金214亿元，增长91%；外贸出口8.1亿美元，增长9.4%。锦州越来越为国内外所关注，沿海区位优势逐渐显现。

　　城镇居民人均可支配收入15350元，增长10%；农民人均纯收入6820元，增长12%。改革发展成果更多惠及百姓，人民群众生活水平不断提高。

　　通过一年的不懈努力，各项主要指标顺利实现“两个不低于”，我们赢得了“四个确保”的重大胜利！

　　（二）基础设施建设实现重大突破，沿海经济带建设取得新成就

　　锦凌水库开工建设，几代人的梦想即将变为现实。滨海大道竣工通车，28.68公里抻直入海工程创造了全国公路建设的奇迹。中电投锦赤铁路、国有铁路扩能改造工程快速推进，滨东大道、阜盘高速锦州段、盘海营客运专线开工建设。大唐锦州热电2台30万千瓦机组、国电黑山10万千瓦风电等一批能源项目投产发电。城市热网改造工程和6座污水处理厂投入试运行。华润锦州发电厂2台100万千瓦机组、城市垃圾发电等一批项目顺利推进。

　　锦州港与大连港深度合作效益显现。209、210集装箱泊位开工建设，西部港区基础工程进展顺利，中电投和华润电力煤炭码头项目已经报批。港口吞吐量完成5200万吨，增长10%；集装箱运输68万标箱，增长4.6%。西海经济区基础设施建设进一步完善。白沙湾行政生活区路网建设、房地产开发、绿化亮化工程快速推进，滨海体育中心、网羽运动中心、职教园区开工建设。娘娘宫经济区规划已经完成，光伏和纺织产业园以及水、电、路等基础设施建设全面展开。大有经济区起步区建设全面开工。沿海经济带“五大板块”建设加速推进，大开发、大开放的发展格局初步形成。

　　（三）实施产业结构优化升级，新兴支柱产业进一步壮大

　　坚持经济结构调整，不断优化三次产业结构。全市三次产业比例由2008年的18∶43.9∶38.1调整为16.7∶44.5∶38.8。

　　工业经济实力显著提升。全市新增规模以上工业企业175户，产值超亿元企业达到200户。光伏、汽车及零部件、精细化工等新兴支柱产业全面发展。晶技太阳能2700万片硅片、日鑫硅1000吨单晶硅、天合精细化工1.5万吨烷基苯磺酸等一批项目竣工投产，阳光能源300千瓦光伏发电站投产并网发电。华昌光伏200MW太阳能电池、博阳光伏科技300MW太阳能电池等一批项目开工建设。科技创新能力不断增强，国家级高新技术企业达到18家，54个项目列入国家、省科技计划。规模以上工业企业高新技术产品增加值实现70亿元，增长20%。节能减排工作进入全国先进行列，万元GDP综合能耗下降5.3%，主要污染物排放量削减5%，城区空气质量优良率达到90%以上。国企改革进一步深化，18户企业完成改制，40户工业企业实行属地化管理。污水处理厂完成股份制改造，燃气总公司顺利实施了股份制改造。

　　农业农村经济稳步增长。农业综合生产能力显著增强，粮、菜、肉、蛋产量均居全省前三位。畜产品质量安全监测位居全国第一。农业产业化水平不断提高，设施农业面积大幅增加，农业标准化进程全面提速，农村生产生活条件进一步改善，新农村建设取得新的进展。

　　县域工业园区建设快速推进。黑山庞河、北镇沟帮子、凌海大有、义县七里河等重点工业园区累计投入基础设施建设资金12.3亿元，实现“五通一平”的建成区面积达到15平方公里。园区主导产业全面确立，产业集群式发展格局初步形成。山东华山集团联合收获机和雨润集团肉食品加工项目落户黑山。投资18亿元的辉山乳业项目落户义县，90万吨焦炭项目正式投产。新增固定资产投资500万元以上企业50户，其中亿元以上9户。县域财政一般预算收入17.6亿元，增长55.3%。黑山、义县财政一般预算收入在全省排位分别上升了4位和3位，北镇、凌海也各上升1位。县域经济的迅猛发展，特别是“义县速度”的脱颖而出，让我们在困难面前显得更加从容和自信。

　　现代服务业发展实现新的突破。老城区改造及中央大街十里商街规划已经完成，招商工作全面展开。锦州国际酒店、文化体育会展中心、千盛购物广场、天宇商务大厦主体工程全部竣工。农村消费市场日益活跃，家电下乡销售8.6万台。新建改造“万村千乡市场工程”连锁农家店200个，覆盖面达到80%以上。完成房地产开发投资49.6亿元，施工面积542.3万平方米。金融部门在经济发展中的作用日益增强，新增各项贷款83.9亿元，增长27.7%。融资担保体系不断完善，6家小额贷款公司开业运营。锦州银行继天津后又在沈阳、大连设立分行。中国(锦州)北方农业新品种新技术展销会、古玩文化节等会展品牌日趋成熟。旅游业实现总收入89.1亿元，增长28.2%。

　　（四）加大招商引资工作力度，对外开放迈出新的步伐

　　充分利用沿海经济带国家战略名片，全方位扩大锦州对外影响。坚持以工业园区为载体，以项目建设为核心，积极创造项目落地条件，精心组织招商引资活动。“十里商街——中央大街改造项目”、“中国海洋城”等主题概念招商影响广泛，锦州光伏、黑山农机装备制造、义县电力电器、北镇工业炉、凌海航天航空新材料和中国北方(锦州)纺织工业城等集群式招商推介活动成效明显。共引进项目155个，其中亿元以上项目43个。

　　加大跟踪服务力度，深挖缴资潜力，重大利用外资项目进资顺利。阳光能源成功在台湾挂牌上市，募集资金2.28亿港元。汉拿电机、万友机械成功并购英属维尔京年城有限公司和香港友桦有限公司。大力实施“十百千高端人才”引进工程，引进海外研发团队项目5个、国外智力项目12个、外国专家230人次。克服金融危机不利影响，促成锦州中石油加工贸易出口大幅增长。继续扩大进出口主体规模，新批自营进出口企业40家。

　　（五）大力实施民生工程，社会事业全面发展

　　就业工作成效显著。城市登记失业率控制在3.7%以内，普惠制就业培训6.1万人，城镇实名制安置就业9.7万人，零就业家庭保持动态为零，登记失业困难家庭大学生全部实现就业。

　　保障体系日趋完善。城镇居民基本养老、医疗、失业、工伤、生育保险参保人数分别达到41.7、112.9、38、26、15万人。城区、城镇和农村最低生活保障标准分别提高20%、32%和26%。城市、农村低保对象分别达到7.96、8.4万人。完成保障性住房实物配租1769套、租赁补贴884户、经济适用住房2500套。

　　扶贫帮困工作取得新的成绩。完成整村推进式扶贫项目37个。3.9万低收入贫困人口的温饱问题稳定解决。救助灾民18.9万人次。发放慈善福彩助学金95.4万元。改造农村低保户危房879户，维修灾民住房1300户。新建农村区域性中心敬老院4所，改造乡镇敬老院9所。

　　文化教育事业全面发展。新建农村九年一贯制学校16所，改造农村中小学危房2.2万平方米，率先在全省完成校舍安全排查鉴定。黑山职教中心主体工程完工。“提高普九”工作全面完成。建成乡镇综合文化站20个、农家书屋400个。京剧《东丹王传奇》等4部作品获省十一届“五个一工程”奖和组织奖。《陈桂秋评剧表演艺术》等4个项目入选省第三批非物质文化遗产保护名录。央视专题片《走遍中国看锦州》在国内外产生广泛影响。开展全民健身活动，建设农村健身示范广场30个。圆满承办了中国国际象棋棋王棋后赛等体育赛事，在第十一届全运会上取得4金3银1铜佳绩。

　　医疗卫生事业稳步推进。基层医疗卫生服务体系建设进一步完善，新建和改扩建县级医院4家、乡镇卫生院11家、社区服务中心7个。完成市传染病院二期工程和市中医院门诊楼建设。老年心理医院正式挂牌。农村常住人口新农合参合率达到100%。防控甲型H1N1流感工作卓有成效。5家新建、扩建的县级计划生育服务站和20家乡级中心服务站投入使用。人口出生率和自然增长率控制在5.68‰和1.51‰。

　　城乡环境明显改观。云飞街小凌河大桥开工建设，东湖森林公园三期工程和松山新区“九街五路”绿化工程全面完成。吉祥街、北京路等18条街路新建、大修、改造工程如期完工。新建、改造路灯1552基，“两河四桥”等城区景观亮化工程顺利完成。加大交通秩序、占道市场、路园环境等整治力度，城市综合整治收到明显成效。新建乡镇客运站10个，1109个村全部通油路。

　　和谐锦州建设扎实有效。公安信息化建设成果在实战中得到凸显，社会治安防控体系日益完善，社区防控能力不断加强，人民群众安全感进一步提升。超额完成法律援助任务，乡镇司法所国债项目全部完成。深入开展“信访积案化解年”和“解难题促和谐”活动，妥善解决了一批突出信访问题。“安全生产年”活动成绩明显，保持了安全生产的平稳态势。通过全市上下的共同努力，圆满完成了国庆安保任务。

　　（六）切实转变机关作风，政府自身建设不断加强

　　自觉接受人大法律监督、政协民主监督和群众的社会监督，坚持定期向市人大和市政协报告、通报工作，认真听取各民主党派、工商联和无党派人士意见。365件代表建议和政协提案全部办结，见面率、办复率达到100%。进一步规范行政执法，政府行政行为步入法制化、规范化轨道。

　　全面开展执法、廉政和效能监察，从严查处各类案件。扎实推进政务公开，公共管理和服务水平进一步提升。积极开展新一轮“思想大解放”和“十学大连”活动，政府系统作风建设有效改善，公务员队伍整体素质不断提高。

　　拥军优属、军民共建活动深入开展，国防动员、民兵预备役工作成绩显著。国家安全、民族宗教、妇女儿童、外事侨务、防震减灾工作不断加强。审计、统计、质监、物价、气象、档案、对台、残联等工作也取得新的进展。

　　各位代表，过去一年的成绩来之不易，面对金融危机带来的冲击和影响，我们经受住了多方面的挑战和考验。一年来，我们戮力同心，负重奋进，发展思路更加清晰明确，精神状态和工作干劲空前高涨，不仅确保了经济的平稳较快增长、民生的进一步改善和社会的持续稳定，而且实现了重大基础设施项目建设的重大突破和三次产业的协调发展，为滨海新锦州建设积聚了强大的发展后劲。实践再一次证明，市委的领导是坚强有力的，“锦州人是能干事的，是能干成大事的”！在此，我代表市政府，向在各个领域和岗位上辛勤劳动、做出贡献的全市人民表示崇高的敬意！向给予政府工作大力支持的人大代表、政协委员、各民主党派、工商联和各人民团体，向关心和支持政府工作的离退休老同志，向中省直各部门、各单位，向驻锦部队和武警官兵，向所有关心支持锦州发展的海内外朋友，表示衷心的感谢！

　　回顾去年的工作，我们也清醒地看到，全市经济社会发展中还面临不少困难和问题。经济实力还不够强，与区域中心城市的要求还不相适应；粗放型发展方式还没有根本转变，经济结构还不尽合理；经济外向度不高，外贸出口压力仍然很大；财政收支矛盾突出，社会保障能力还十分有限；涉及群众切身利益的一些问题还没有完全解决，不稳定因素仍然存在；软环境建设还存在一些不容忽视的问题，政府自身建设有待进一步加强，等等。这些困难和问题，需要我们在今后的工作中切实加以解决。

　　二、2010年工作安排

　　2010年，是“十一五”规划的最后一年。做好今年工作，对于全面推进锦州经济社会发展、实现“三年倍增”计划和“十二五”规划的高起点开局尤为重要。今年全市经济社会发展的总体要求是：全面贯彻落实中央、省经济工作会议和市委十届八次全会要求，以科学发展观为指导，积极抢抓机遇，深入实施“一纲五线”发展战略，更加注重经济发展方式转变和结构调整，更加注重改革开放和自主创新，更加注重节能减排和生态建设，更加注重改善民生和社会稳定，继续保持经济社会又好又快发展态势，全面完成“十一五”规划任务，努力为“十二五”实现新跨越奠定坚实基础。

　　今年经济社会发展的主要预期目标是：地区生产总值增长17%，达到915亿元；地方财政一般预算收入增长21%，达到58亿元；全社会固定资产投资增长60%，达到560亿元；社会消费品零售总额增长22%，达到330亿元；实际利用外资和域外资金分别增长36%和40%，达到5亿美元和300亿元；外贸出口增长15%，达到9.3亿美元；城镇居民人均可支配收入和农民人均纯收入分别增长11%和12%。

　　以上指标，与“三年倍增”计划和建设辽西沿海经济区中心城市的总体目标相衔接，体现了科学发展、和谐发展、跨越发展的要求。在实际工作中，我们将向更高的目标努力，力争更好更快发展，确保主要指标增幅不低于全省平均水平、总量不低于去年在全省的位次。

　　为实现上述目标，我们要做好以下七项工作：

　　（一）加快沿海经济带重大基础设施建设

　　以沿海经济带基础设施建设为突破口，加快推进锦州港、西海经济区、娘娘宫经济区、大有经济区、建业经济区“五大板块”开发建设，努力形成组团开发、横向连接、互为支撑的沿海经济带发展新格局。全市共安排亿元以上基础设施项目48个，总投资609亿元。

　　加快亿吨大港建设。继续推进锦赤铁路通道扩能等项目建设，确保中电投和华润电力煤炭码头项目年内开工，209、210泊位竣工交付使用。完成港建投资19.8亿元，其中新建10.2亿元。港口吞吐量达到5500万吨，集装箱运输70万标箱。

　　加快西海经济区建设。进一步完善标准厂房等配套设施建设，确保石油储备基地、蒙古贞热电厂、15万吨高碳铬铁等18个重点项目如期开工。全面完成白沙湾行政生活区路网及水、电等配套设施建设，重点打造沿海景观带和中轴线景观带。加大汤河子工业园区基础设施建设力度，不断增强产业集聚功能。

　　加快娘娘宫经济区建设。6.2平方公里商住起步区建设全面启动。光伏产业园实现“七通一平”，已落户的5个项目竣工投产。着手筹办世界园艺博览会。抓紧启动娘娘宫新港建设规划。开工建设城际铁路。滨海大道绿化、亮化工程全面完成，滨东大道竣工通车。

　　加快大有经济区建设。进一步完善经济区发展规划。大力推进航天航空新材料产业园配套设施建设，确保5平方公里起步区具备项目落地条件。开工建设锦州机场和滨海大道至凌海支线。

　　加快建业经济区建设。抓紧启动经济区、锦州湾航空物流城发展规划编制工作和起步区基础设施建设。

　　协调推进腹地能源、交通、农业、商贸流通、文化教育、医疗卫生等方面的基础设施建设，努力为沿海经济带开发开放创造更为有力的腹地支撑。

　　（二）确保工业经济稳定增长

　　积极推进工业产业结构调整，在发展壮大石化、铁合金、农产品深加工等传统支柱产业的同时，进一步加大光伏、汽车及零部件、精细化工等新兴支柱产业培育力度，加快实现产业集群化发展。全年共安排亿元以上重点工业项目46个，确保规模以上工业增加值增长26%，工业固定资产投资增长30%。

　　加快特种铁合金产业发展。确保中信铁合金30万吨中低碳锰铁扩能改造、天桥钼业3500吨精深加工等项目建成投产，中信铁合金1万吨电解金属镁、2.7万吨氯气、1万吨海绵钛和宝钛华神6000吨海绵钛技术改造等项目开工建设。实现铁合金总产量85万吨、产值280亿元。

　　加快光伏产业发展。确保新世纪1000吨低成本多晶硅、锦懋光伏科技200MW太阳能组件、博阳光伏科技600MW太阳能组件、佑华硅2000吨单晶硅及8000万片硅片等5个项目建成投产，推进新世纪1万吨低成本多晶硅、佑华硅2000吨多晶铸锭、博阳光伏科技200MW多晶铸锭及切片等7个项目建设进程，全面启动光伏并网发电、太阳能光电建筑应用示范和光伏发电城市亮化工程。实现光伏产业产值100亿元，增长230%。

　　加快汽车及零部件产业发展。确保锦恒40万套方向盘、14万套安全气囊产业化技术改造和抚挖锦重460台大吨位汽车起重机、全路面起重机技术改造等项目建成投产，恒亚内燃机配件16万支曲轴扩产技改等项目开工建设。实现整车和汽车零部件产值90亿元，增长80%以上。

　　加快精细化工产业发展。确保锦州石化公司130万吨加氢裂化装置、天合精细化工2万吨无灰分散剂等项目竣工投产，中信铁合金3万吨钛白粉、锦州石化公司100万吨催化汽油脱硫装置、10万吨异丙醇装置等项目开工建设。实现精细化工产值45亿元，增长30%以上。

　　认真做好节能减排工作。确保万元GDP综合能耗降低5%，主要污染物排放量削减5%，城区空气质量优良率达到90%以上。

　　（三）发展壮大县域经济

　　加快县域工业园区基础设施建设，推进项目向先导区集中，向特色主导产业集聚。各工业园区起步区都要实现“七通一平”，新建标准厂房30万平方米以上，落户亿元以上工业项目40个以上，确保每个县(市)尽早形成一个超百亿元的工业产业集群。辉山乳业液态奶项目正式投产，饲养3.36万头奶牛的12个养殖场全部建成投入使用。再掀县域经济发展的新高潮，努力实现财政一般预算收入和农民人均纯收入更大幅度的增量升位。

　　大力发展现代农业，加快构建五大产业结构和五大运营体系，推进农业产业化向纵深发展。新增规模以上产业化龙头企业50家以上、设施农业面积20万亩、水产健康养殖示范区23个。全面推进畜牧大市向畜牧强市转变，新建畜禽标准化养殖小区300个。引进推广农业新品种、新技术100项，新建千亩以上农业科技示范园区50个，标准化生产面积达到500万亩，蔬菜水果等农产品检验检测率达到50%以上。农业机械化水平达到65%。新建农民专业合作社100个，转移农村剩余劳动力5万人。

进一步改善农村生产生活条件，确保新农村建设取得新的成效。加强气象防灾减灾能力建设。加大基本农田和土地开发保护力度，确保耕地保有量继续实现正增长。大力推广畜牧业生态养殖技术，扎实推进重大动物疫病防控、畜牧业投入品及畜产品质量安全保障体系建设。继续实施三年绿化工程，切实抓好南山绿化三期、锦凌水库水源涵养林等8大重点生态建设工程。建设1.2万个户用沼气池、2处省级秸秆气化站。解决农村6万人的安全饮水问题。

　　（四）加快城区经济发展

　　借鉴县域经济“双增升位”的成功做法，强力推进城区经济发展。进一步完善城区经济社会管理职能，最大限度地下放管理权限。加快推进娘娘宫“凌古”工业园区规划和基础设施建设，加大项目引进力度，切实解决老城区发展空间受限问题。按照主要经济指标三年翻一番的奋斗目标，科学制定考核评价体系，确保城区地区生产总值增长35%以上，地方财政一般预算收入增长50%以上。

　　围绕中央大街十里商街重点项目建设，高标准启动老城区的开发改造，确保亿隆国贸、温州名购、茂业百货、家乐福商场等一批商业项目开工建设。加快推进香港锦昌、深圳大中华、山西宝地置业等在锦投资项目进程，尽快提升滨海新区服务业发展水平。继续推进农村商品流通网络和“万村千乡市场工程”建设，增强农村消费拉动能力。整合旅游资源，重点推进“大闾山”开发建设，加快打造滨海大道旅游观光带。安排房地产项目110个，总投资126亿元，建筑面积638万平方米。积极推进企业上市工作。大力发展非银行业金融机构和中介组织，全市小额贷款公司发展到20家。继续加大对政府关联类项目和重点工业项目的支持力度，银行信贷资金实现倍增。

　　（五）强力推进对外开放

　　抢抓国家战略机遇，精心包装策划，开展强势宣传，全面掀起新一轮对外开放热潮。坚持以沿海经济带产业园区和县域工业园区建设为平台，认真组织好一月底香港、四月大连、六月和九月长三角、珠三角等招商活动。举全市之力，切实抓好电力电器、工业炉、农机装备制造、航天航空新材料、光伏、汽车及零部件、精细化工、商业地产等主导产业的集群式招商，确保在引进规模大、技术高、产业关联度强的重大项目上实现重大突破。依托现有国企资源，积极寻找战略投资者，盘活资产，推进改革。

　　用足用好国家、省鼓励出口政策和资金，完善服务体系，提高服务水平，竭力为出口企业创造有利条件。重点做好油品加工贸易，保持出口规模。进一步调整出口产品结构，积极开拓机电、光伏等高技术含量、高附加值产品的国际市场，确保外贸出口不低于全省平均水平。

　　（六）加快以改善民生为重点的社会事业发展

　　加大就业工作力度。强化与市场对接的普惠制培训和创业培训，完成普惠制培训3.5万人，创业培训1500人，扶持创业带头人310人。确保完成城镇实名制就业5.2万人，零就业家庭保持动态为零，登记失业困难家庭大学生全部实现当年就业，城镇登记失业率控制在4.5%以内。

　　完善社会保障体系。全面启动新型农村养老保险试点工作，开展保险扩面专项行动，确保政策覆盖范围内的人群得到应有保障。提高城市低保对象分类救助标准，农村低保标准提高到1440元。切实解决25万农村受灾群众基本生活问题。新增城镇养老床位400张。新增城市社区办公用房5000平方米，完成100个农村社区服务中心建设任务。新建廉租住房1769套，新增租赁补贴1348户，建设经济适用住房2500套。

　　加强文化教育建设。推进义务教育均衡发展，深入实施“双高普九”规划。新建农村九年一贯制学校11所，改造农村中小学危房2万平方米，完成市职教园区一期工程。加强乡镇中心幼儿园建设。积极推进文化体制改革。继续开展笔架山海洋音乐节、国际民间文化节、古玩文化节、古塔庙会、周末文化广场等文化活动。如期完成“农家书屋”建设。深入实施数字电视整体平移工程。精心组织参加省第十一届运动会，全力承办好省第七届少数民族运动会。

　　推进医疗卫生工作。认真做好医药卫生体制改革，开展乡镇卫生院与城市大型医院集团化经营管理试点。强化城市社区卫生服务体系建设。打造全市医疗信息共享平台，建立覆盖城乡的三级急救网络体系。确保基本公共卫生服务经费年人均不低于15元。新农合筹资标准由年人均100元提高到150元。大力推进家庭健康促进计划，全面落实计划生育家庭特别扶助和农村部分计划生育家庭奖励扶助政策。

　　提升城乡建设管理水平。加快推进锦凌水库建设。实施站前广场改造。新建、大修云飞南街、解放路等11条街路，完成50条街路人行步道的修建任务。增改交通信号灯126处、视频监控系统216处。广州街公铁立交桥、中环路三期等工程开工建设，云飞街小凌河大桥建成通车。启动松山新区欧洲风情小镇建设。完成凌南供热管网改造。新增集中供暖面积50万平方米。更新改造295栋住宅楼老旧二次供水管线及设施。新增燃气用户1万户。实行城市管理相对集中处罚权，巩固整治成果，提升管理标准。大力推进县城老城区改造和小城镇建设试点。新建乡镇客运站7座。完成滨东大道等8项交通工程。

　　加快和谐锦州建设进程。巩固军政军民团结，争创全国双拥模范城“三连冠”。认真开展第六次人口普查。高水平编制好“十二五”规划。完成“五五”普法任务，做好“六五”普法准备。深化公安信息化建设，完善社会治安防控体系，加强城市公共安全视频监控系统建设和管理，提高应急处置能力和水平，加快推进平安锦州建设。创新信访工作机制，全面完成市县两级信访大厅建设任务，实现信访工作投诉在大厅、办理在大厅、解决在大厅。严格落实信访工作责任制，加强行政复议工作，有效化解各类社会矛盾。强化行政领导责任和企业主体责任，加强重点领域、重点行业安全隐患的排查和整治，高度重视食品、药品、交通和消防安全，坚决遏制重特大事故发生。

　　（七）切实加强政府自身建设

　　坚持依法行政。自觉接受人大法律监督、工作监督和政协的民主监督，继续坚持重大事项向人大报告、向政协通报制度，严格执行人大决议、决定，积极支持政协履行政治协商、参政议政职能，认真办理好人大代表议案、建议和政协提案。

　　提高行政效能。深入实施政务公开，推进决策公开民主。深化行政审批制度改革，严格落实《服务承诺制》、《首问责任制》、《限时办结制》、《行政处罚自由裁量权暂行规定》等规章制度。加快推进政府公共服务平台建设，进一步优化经济发展环境。

　　加强廉政建设。认真落实党风廉政责任制，严格执行廉洁自律各项规定。深入开展行政监察、审计监督和纠风专项治理行动，坚决纠正阻碍发展和损害人民群众利益的不正之风，进一步规范各级政府和公务人员的行政行为。

　　转变工作作风。大力弘扬“勇于牺牲，敢为人先”的锦州精神，自觉践行“科学加拼命”的工作作风，扑下身子，直面困难，聚精会神搞建设，一心一意谋发展，奋力开创各项工作的新局面。

　　各位代表，我们正面临着千载难逢的机遇和前所未有的挑战。让我们在市委的正确领导下，紧紧依靠全市人民，不畏艰险、一往无前，抢抓机遇、乘势而上，为全面推进锦州沿海经济带开发开放，加速辽西沿海经济区中心城市建设而努力奋斗！
